# Bifunctional Cysteine-Engineered CAR‑T Cells Enable Thiol-Mediated Targeting to Overcome Antigen Escape in B Cell Lymphoma

**DOI:** 10.1021/acscentsci.5c00816

**Published:** 2025-08-07

**Authors:** Jost Lühle, Simon Krost, Felix Goerdeler, Aina Valentí, Elena Shanin, Christian Seitz, Peter H. Seeberger, Oren Moscovitz

**Affiliations:** † Department of Biomolecular Systems, 28321 Max Planck Institute of Colloids and Interfaces, 14476 Potsdam, Germany; ‡ Institute of Chemistry and Biochemistry, Freie Universität Berlin, 14195 Berlin, Germany; § Department of Pediatric Hematology and Oncology, 9188 University of Tübingen, 72074 Tübingen, Germany; ∥ Hopp-Children’s Cancer Center Heidelberg (KiTZ), 69120 Heidelberg, Germany; ⊥ Department of Pediatric Oncology, Hematology and Immunology, Heidelberg University Hospital, 69120 Heidelberg, Germany

## Abstract

Chimeric antigen receptor (CAR) T cell therapy has revolutionized the treatment of hematologic malignancies; however, durable remissions remain limited due to antigen-negative cancer relapse, where tumor cells downregulate or lose the targeted antigen to evade immune recognition. To address this challenge, we developed cysteine-engineered CAR (CysCAR) T cells that redirect T cells to target cancer cells based on extracellular redox imbalances and the altered thiol/disulfide ratios, a marker we identified on B cell lymphomas. Here, we show that CysCAR-T cells, engineered with different cysteine-modified antibody fragments, exhibit a potent and specific cytotoxicity *in vitro* across various B cell lymphoma (BCL) subtypes, even in antigen escape models. Moreover, by integrating cysteine engineering with clinically used anti-CD19 CAR-T cells, we enabled simultaneous targeting of CD19 and altered redox states on BCL, potentially reducing the risk of antigen escape. In a pilot *in vivo* study, these bifunctional CD19-CysCAR-T cells suppressed tumor growth and prolonged survival of BCL-bearing mice without inducing systemic toxicity. Given that aberrant exofacial redox states are a hallmark of multiple cancers, our findings suggest a promising strategy to enhance the efficacy of anti-CD19 CAR-T cell therapy, overcome antigen escape, and reduce tumor relapse in BCL, with potential applicability to other malignancies.

## Graphical abstract

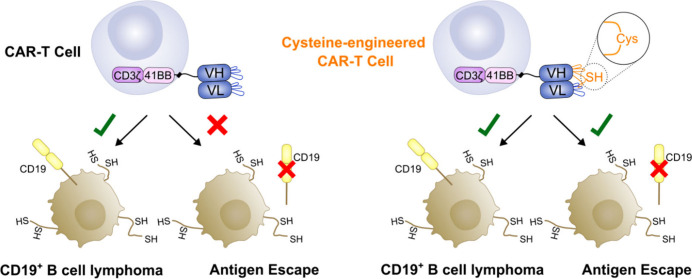

## Introduction

The adoptive transfer of T lymphocytes engineered with CD19-specific chimeric antigen receptors (CAR) has transformed the therapy of B-cell lymphoma (BCL) and other hematological cancers.^1,2^ However, up to 25% of BCL patients relapse or fail to respond to the therapy after the treatment.^3^ Among the most pressing therapeutic resistance mechanisms is antigen escape, where selective pressure from CD19-targeted CAR-T cell therapy leads to the downregulation or loss of CD19 expression on BCL cells.^4,5^ Thus, additional strategies that target antigen escape in cancer are necessary to diminish tumor relapse and achieve sustained, long-term remission.

Ongoing preclinical and clinical research focuses on improving CAR-T cell function. CAR designs have evolved significantly in recent years to sustain and amplify the effectiveness of CAR-T cells. In particular, the second-generation CAR-T cells featuring a CD3ζ signaling domain and a single costimulatory domain (CD28 or 4-1BB) marked a turning point in clinical success.^6^ Building on this foundation, next-generation CAR-T cells integrate advanced features ranging from costimulatory domains and cytokine-secreting functions to inducible gene circuits designed to enhance CAR-T cell persistence, adaptability, and tumor infiltration.^7,8^ Moreover, some of those focus on co-targeting additional tumor-associated antigens (TAA), such as CD20, or employing bispecific or adapter-based CAR designs to overcome CD19-specific resistance.^9–11^ However, these approaches still rely on specific TAAs, allowing for further cancer adaptation.

An emerging strategy to enhance CAR-T cell effectiveness involves engagement with the extracellular components of the tumor microenvironment, thereby broadening the therapeutic potential and reducing the risk of immune evasion.^12^ Among the defining features of cancer cells is their altered redox state,^13^ which presents a novel opportunity for CAR-T cell treatment. Traditionally, redox dysregulation in tumors has been attributed to elevated levels of reactive oxygen species (ROS), which contribute to genomic instability and cancer progression. However, increasing evidence suggests a more complex and dynamic situation, where an adaptive upregulation of antioxidant systems contributes to abnormal redox states that differ in a highly localized and specific manner.^14–16^ In this model, tumor cells enhance their antioxidant responses through differential expression of disulfide-active enzymes, such as protein disulfide isomerases (PDI)^17^ and components of the thioredoxin system (Trx),^18^ which are both partially exported to the extracellular space.^19–21^ Together, these adaptations shift the redox balance of the cell surface, leading to altered thiol/disulfide ratios on the membranes of cancer cells, including BCL.^22,23^ This redox remodeling enables cancer cells to buffer intracellular oxidative stress and contributes to the formation of an immunosuppressive microenvironment that supports inflammation, tumor growth, and therapy resistance.^24^

Several studies have underscored the importance of extracellular redox adaptation in cancer. For instance, elevated levels of reduced thiol groups on breast cancer cells have been shown to adhere more effectively to the endothelium, thereby promoting metastasis.^25^ Similarly, PDI upregulation has been linked to increased proliferation and drug resistance in various cancers.^26–28^ Notably, pharmacological intervention of PDI has been shown to prevent metastasis and improve survival in preclinical cancer models,^29,30^ highlighting the therapeutic potential of targeting the reduced tumor cell surface. However, this redox-based vulnerability remains unexplored in CAR-T cell therapy, likely due to the lack of molecular tools that enable precise recognition of the altered redox state of cancer cells.

Previously, we reported the development of a redox-specific single-domain nanobody we termed CB2.^23^ This nanobody specifically recognizes and binds to altered redox states on the surface of the BCL cells. Its specificity is conferred by a noncanonical cysteine 105 in the complementarity-determining region (CDR) 3, which enables binding to multiple targets on BCL cells exhibiting an imbalanced thiol–disulfide equilibrium. This mechanism allows CB2 to serve as a redox sensor, making it a powerful tool for probing, modulating, and targeting the redox landscape of BCL cells. It is hypothesized that recognition of target cells by redox-sensitive compounds, such as CB2, involves thiol–disulfide exchange reactions at the cell surface, forming transient and reversible covalent bonds with exofacial thiols that become exposed under altered redox conditions commonly found in cancer.^31,32^

Here, we report a next-generation cysteine-engineered CAR (CysCAR) T cell platform that enables targeting of the BCL cell surface redox environment rather than a single antigen. In this pilot study, we evaluate the preliminary efficacy and feasibility of cysteine-engineered CAR-T cells in a murine antigen escape model, providing initial evidence of their therapeutic potential. Cysteine-engineered CAR-T cell antibody fragments enable their activation and cytotoxicity across multiple subtypes of BCL *in vitro*, while sparing healthy cells, underscoring their selectivity and safety. Moreover, we show that cysteine-engineering of state-of-the-art anti-CD19 CAR-T cells enables them to co-target conventional antigens (CD19) and altered redox states simultaneously, potentially reducing the risk of antigen escape. By defining key optimization criteria for cysteine-engineered bifunctional CAR constructs, we establish a universal and adaptable framework for next-generation CAR-T therapies with enhanced resilience against antigen escape and broader therapeutic applicability.

## Results and Discussion

### CB2 Cysteine-Engineered CAR-T Cells Specifically Eliminate BCL while Sparing Healthy Lymphocytes

With the goal of developing CysCAR-T cell therapy that could target the extracellular redox microenvironment of cancer cells in a cysteine-specific manner, we used CB2 nanobodies previously demonstrated to interact with BCL cells through a thiol-based mechanism.^23^ Building on this finding, we envisioned the potential of CB2 as a guidance system for directing CAR-T cells toward BCL in a thiol-dependent manner (hereafter, CB2-CAR, Figure 1A).

**Figure 1:**
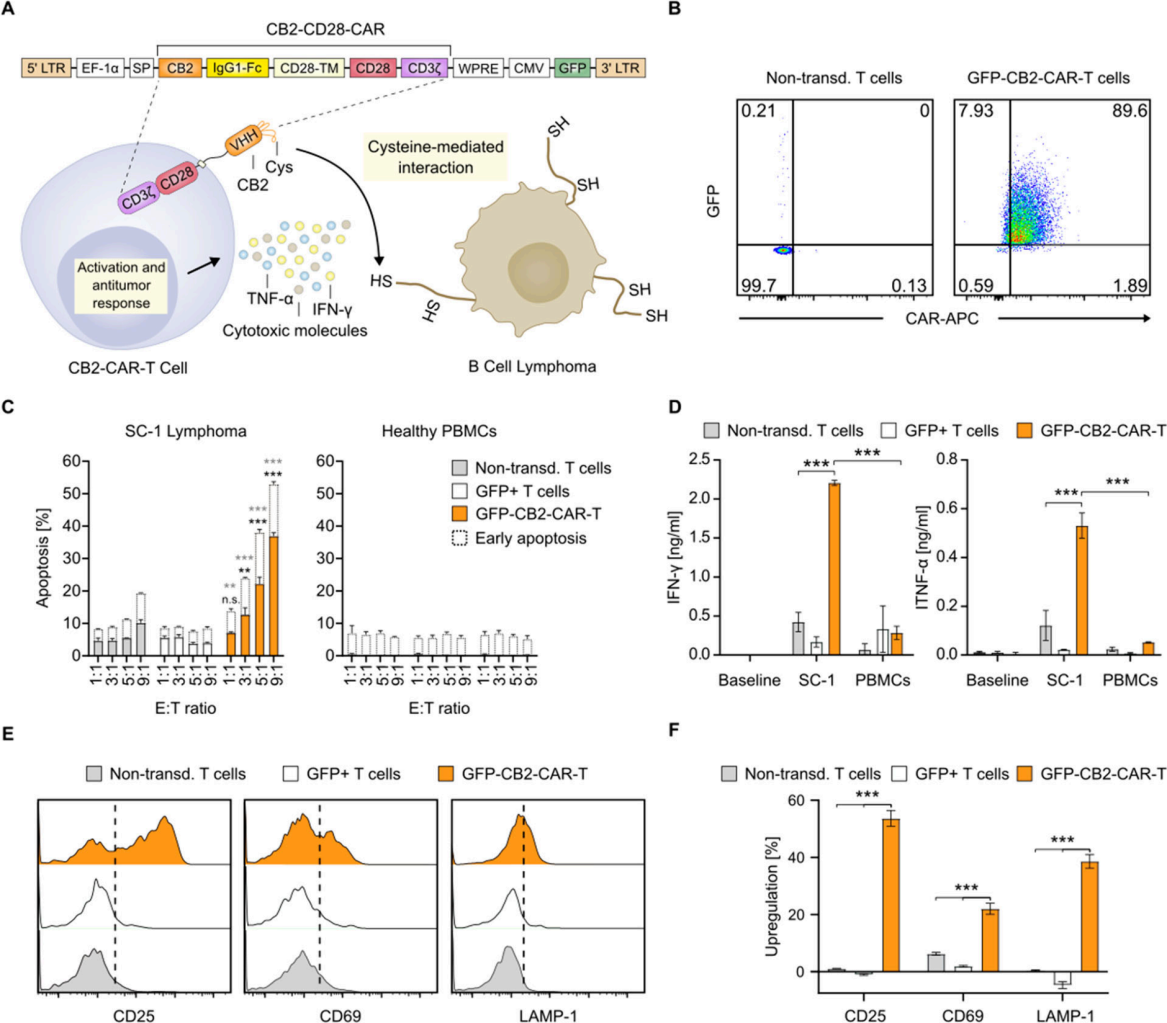
CB2-CAR-T cells exhibit cytotoxicity against B cell lymphoma and undergo T cell activation. (**A**) Schematic of CB2-CD28-CAR construct with GFP (*upper panel*) and a schematic of cysteine-dependent CB2-CAR-T cell activity against BCL (*bottom panel*). (**B**) Representative flow cytometry analysis of sorted GFP-CB2-CAR-T cells to determine the transduction efficiencies of CAR-expressing T cell on day 6. (**C**) Apoptosis assay of SC-1 cells or healthy PBMCs cocultured with CAR-expressing cells (*bottom bars*: late apoptosis, *top bars*: early apoptosis). (**D**) Quantification of IFN-γ and TNF-α production by CAR-expressing T cells cocultured with SC-1 cells or healthy PBMCs, as well as T cells alone (baseline). (**E**) Staining of CAR-expressing T cells with CD25, CD69, and LAMP-1 after coculturing with SC-1 cells. (**F**) Quantification of T cell activation marker expression. Data are shown as mean ± SEM of (**C, D**) *n* = 3, (**F**) *n* = 3. Statistical significance was calculated via (**C, D**) two-way ANOVA and Tukey’s *posthoc* and (**F**) one-way ANOVA and Tukey’s *posthoc* tests: n.s., not significant; *p < 0.05; **p < 0.01; ***p < 0.001.

To assess this, we engineered a second-generation CAR construct containing CB2 as the antigen-binding fragment, a human IgG1-Fc as the hinge region, and CD28 serving as both transmembrane and costimulatory domains. Additionally, GFP was included in the same vector as a reporter protein (CB2-CD28-CAR_GFP, hereafter GFP-CB2-CAR, Figure 1A, upper panel). Using a lentivirus carrying either this construct or only GFP (Figure S1B), we successfully transduced T cells isolated from healthy human PBMCs. GFP-CB2-CAR and GFP+ T cells were then enriched through fluorescence-activated cell sorting, ensuring a highly purified population (Figure 1B, Figure S2).

Next, we validated the functional activity of engineered GFP-CB2-CAR and GFP+ T cells against a BCL cell line model, SC-1 follicular lymphoma, which was previously shown to exhibit an abnormal cell surface redox state.^23^ Notably, GFP-CB2-CAR-T cells exhibited significant concentration-dependent cytotoxicity against BCL > 50% at a 9:1 effector-to-target (E:T) ratio compared to nontransduced or GFP+ T cells (Figure 1C, left panel, Figure S3). In contrast, cocultures with healthy human PBMCs showed no detectable cytotoxic activity (Figure 1C, right panel), suggesting the specificity of the engineered GFP-CB2-CAR-T cells for BCL cells.

To evaluate the effector function of GFP-CB2-CAR-T cells, we measured pro-inflammatory cytokine release and the expression of T cell activation markers as indicators of T cell response upon target cell engagement. For this, we used the enzyme-linked immunosorbent assay (ELISA) to quantify the secretion of IFN-γ and TNF-α upon coculture with SC-1 cells and healthy human PBMCs. In agreement with the apoptosis assay, GFP-CB2-CAR-T cells showed a robust inflammatory response upon SC-1 cell engagement, secreting 4-fold higher levels of both cytokines compared to nontransduced and GFP+ T cells, while healthy PBMCs did not induce cytokine secretion (Figure 1D). Notably, flow cytometry results revealed that the key T cell activation markers CD25 and CD69 and the degranulation marker LAMP-1 were significantly upregulated in the case of GFP-CB2-CAR-T cells cocultured with SC-1 lymphoma cells (Figure 1E), with increases of 54%, 22%, and 39% for CD25, CD69, and LAMP-1, respectively (Figure 1F). Thus, GFP-CB2-CAR-T cells showed robust activation and degranulation.

Taken together, these results confirm that CB2 cysteine-engineered CAR-T cells not only exhibit strong cytotoxic activity but also mount a robust inflammatory response upon target cell engagement. The significant increase in pro-inflammatory cytokines (IFN-γ, TNF-α) and upregulation of T cell activation markers, coupled with the absence of cytokine release in healthy PBMC cocultures, highlights the safety and specific activation of CB2-CAR-T cells. Thus, this underscores the potential of cysteine-engineered CAR-T cells to target BCL with minimal off-tumor effects.

### Cytotoxic Activity of CB2 Cysteine-Engineered CAR-T Cells Depend on Cysteine-Mediated Interactions

The promising activity observed with CB2-CAR-T cells prompted us to investigate whether cysteine engineering could confer functional activity against BCL when incorporated into an unrelated antibody fragment-based CAR. To test this, we employed the anti-GFP nanobody LaG-16 ^33^ as a proof-of-concept. We engineered two CAR constructs: wild-type LaG-16 and its serine-to-cysteine mutant at position 106 (^S106C^LaG-16) and compared these to CB2 and a cysteine-to-serine mutant of CB2 (^C105S^CB2), which was previously shown to abolish CB2 binding upon substitution of cysteine with a serine residue (Figure 2A).^23^ All CB2 and LaG-16 constructs were expressed in *E. coli*, and purified nanobodies were tested for binding to SC-1 cells by flow cytometry (Figure S4). As expected, LaG-16 lacking a cysteine residue did not bind to SC-1 cells, whereas the cysteine-engineered construct ^S106C^LaG-16 showed a robust SC-1 cell binding, mirroring the binding pattern of wild-type CB2 (Figure 2C).

**Figure 2:**
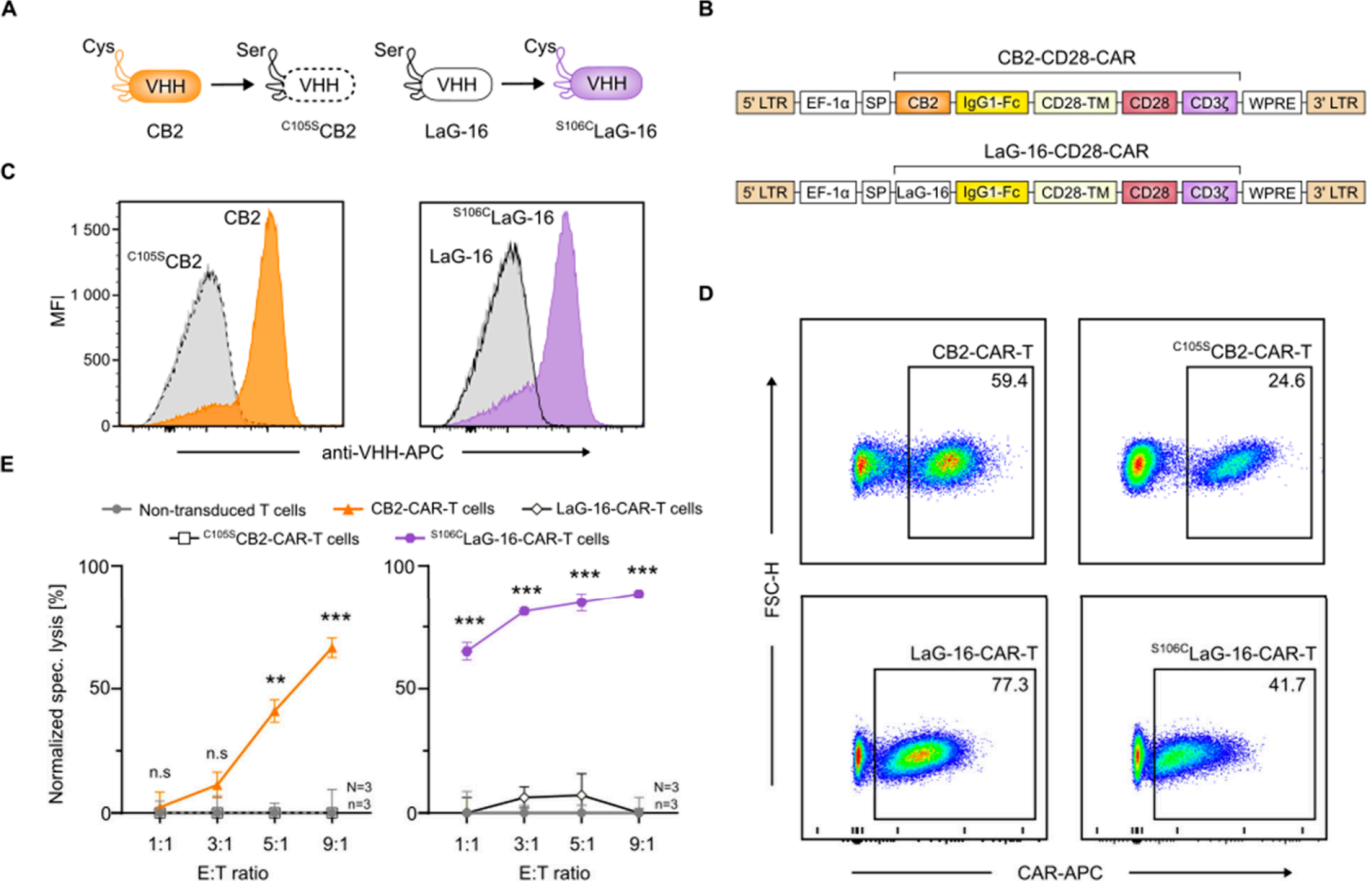
Cysteine-engineered nanobodies redirect CAR-T cells to target BCL. (**A**) Cartoon representation of nanobodies CB2 and LaG-16 and their mutations C105S and S106C, respectively. (**B**) Domain structures of CB2-CD28-CAR and LaG-16-CD28-CAR constructs. (**C**) Flow cytometry binding assay of nanobody or secondary antibody alone as control (*gray*) to SC-1 cells (*n* = 3). (**D**) Flow cytometry analysis of CAR-T cell transduction efficiencies for CB2-CD28-CAR-T (WT or C105S) and LaG-16-CD28-CAR-T (WT or S106C) cells on day 6. (**E**) Specific lysis of SC-1 cells after co-incubation with CAR-T cells for 24 h. Data are shown as the grand mean ± pooled SEM (*N* = 3, *n* = 3). Statistical significance was calculated via two-way ANOVA and Tukey’s *posthoc* test: n.s., not significant; *p < 0.05; **p < 0.01; ***p < 0.001.

Encouraged by these results, we hypothesized that CB2-CAR-T cell activity is solely driven by the presence of a cysteine residue in CDR3 and that incorporating this feature into the antibody fragment could redirect CAR-T cells to the target BCL. To test this, we generated four types of CAR-T cells expressing LaG-16, ^S106C^LaG-16, CB2, or ^C105S^CB2 (Figure 2B–D). As transduction efficiencies varied between constructs (Figure 2D), cell numbers were adjusted in all assays to ensure equivalent effector cell counts. Next, we compared the functional activity of cysteine-engineered ^S106C^LaG-16-CAR- to CB2-CAR-T cells and their respective controls LaG-16-CAR- and ^C105S^CB2-CAR-T cells, using a luciferase-based cytotoxicity assay of cocultures with SC-1 cells. In agreement with the cell binding assays, both CB2-CAR- and ^S106C^LaG-16-CAR-T cells exceeded 50% cytotoxicity at a 9:1 E:T ratio (Figure 2E). In contrast, introducing the cysteine-to-serine mutation in ^C105S^CB2 construct abolished CAR-T cell activation in SC-1 cocultures, confirming that cysteine-mediated interactions are essential for driving cytotoxicity.

Taken together, these results highlight the role of cysteine in enabling CB2 cysteine-engineered CAR-T cells to recognize and eliminate BCL. Substituting cysteine with a serine completely abolished the CB2-CAR-T cell activity. In contrast, the introduction of cysteine into LaG-16-CAR-T cells enabled their activity, highlighting the essential function of cysteine in mediating cancer cell engagement and the cytotoxicity of CysCAR-T cells. Interestingly, the superior performance of ^S106C^LaG-16-CAR-T cells compared to CB2-CAR-T cells points to functional differences among CysCAR-T cells that are influenced by the position of cysteine within the antibody fragment sequence. These findings suggest the critical impact of cysteine positioning in enabling antigen-independent targeting and shaping the overall CysCAR-T cell efficacy.

### CB2 Cysteine-Mediated CAR-T Cell Targeting Eliminates Anti-CD19 CAR-T Cell Therapy-Resistant BCL

We inspected the therapeutic scope of cysteine-engineered CAR-T cells against BCL antigen escape models and compared it to the clinical state-of-the-art CD19-CAR-T cell therapy Kymriah. To match the clinically used Kymriah format (CD19-411B-CAR), we changed the receptor configuration of the CB2 construct (CB2-411B-CAR, hereafter CB2-CysCAR) by introducing costimulatory 4-1BB- and CD8-derived hinge and transmembrane domains (Figure 3A).

**Figure 3:**
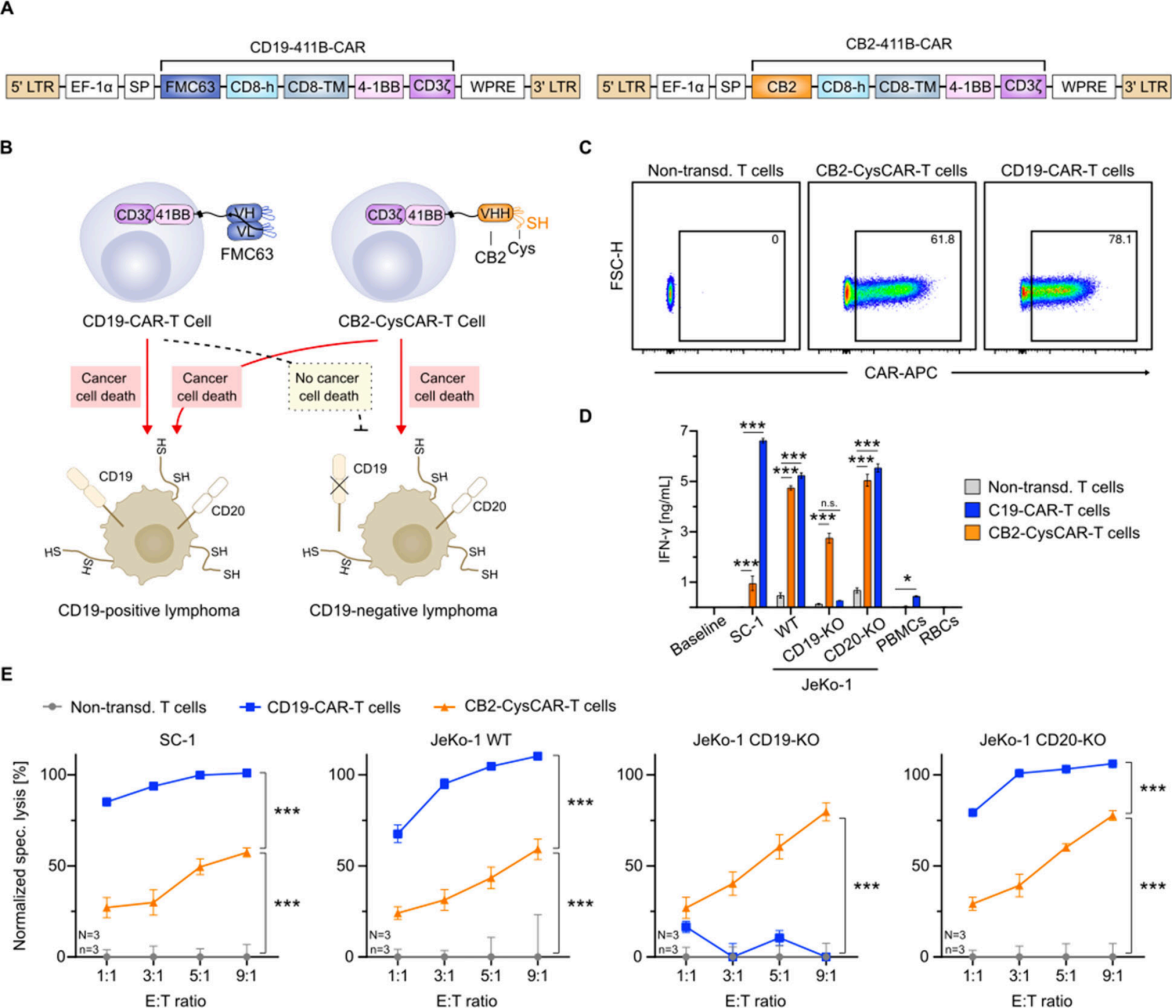
CB2-CAR-T cells efficiently target B cell lymphoma subtypes and antigen escape models. (**A**) Domain structures of CD19-41BB-CAR and CB2-41BB-CAR used to engineer conventional and CB2-CysCAR-T cells, respectively. (**B**) Schematic of bifunctional BCL targeting of CB2-CysCAR-T cells compared to conventional CAR-T cells. (**C**) Representative density maps of conventional and CB2-CysCAR-T cells used to determine T cell transduction efficiencies on day 6. (**D**) Quantification of BCL-induced IFN-γ production by CAR-expressing T cells or T cells alone (baseline) upon coculture with BCL cells (SC-1 and JeKo-1) and PBMCs or RBCs as negative controls. (**E**) Specific lysis of various BCL cells after co-incubation with different primary CAR-T cells. Data are shown as (**D**) mean ± SEM (*n* = 3) and (**E**) grand mean ± pooled SEM (*N* = 3, *n* = 3). Statistical significance was calculated via two-way ANOVA and Tukey’s *posthoc* tests: n.s., not significant; *p < 0.05; **p < 0.01; ***p < 0.001.

First, we generated conventional CD19-CAR- and cysteine-engineered CB2-CysCAR-T cells with high efficiencies of >60% by lentiviral transduction and compared their cytotoxic functionalities against BCL and its antigen escape models (Figure 3B,C). For this, we cocultured them with SC-1 cells and antigen escape models, including JeKo-1 mantle cell lymphoma cells and JeKo-1 CD19- or CD20-knockout variants (Figure S5), and assessed the IFN-γ secretion using ELISA. We included healthy PBMCs and red blood cells (RBCs) as negative controls. In accordance with our previous results, CB2-CysCAR-T cells showed a significant increase in the level of IFN-γ secretion against all cancer cell lines. Notably, IFN-γ levels against CD19-negative cells reached approximately 3 ng/mL, while there was no secretion from conventional anti-CD19 CAR-T cells (Figure 3D). Coculture with healthy PBMCs did not elicit IFN-γ secretion from CB2-CysCAR-T cells, indicating a lack of off-tumor activation. In contrast, conventional anti-CD19 CAR-T cells slightly secreted IFN-γ, likely due to recognition of healthy B cells within the PBMC population.^34^

To demonstrate the clinical relevance of cysteine-engineered CB2-CAR-T cells for targeting antigen escape cancer models, we generated CB2-CysCAR-T cells from three independent blood donors (*N* = 3, *n* = 3). Compared with conventional CD19-CAR-T cells, CB2-CysCAR-T cells exhibited robust, concentration-dependent cytotoxicity above 50% in luciferase-based killing assays against all BCL subtypes. Remarkably, CB2-CysCAR-T cells effectively eliminated 80% of JeKo-1 CD19-knockout cells, which were resistant to the clinically used anti-CD19 CAR-T cells (Figure 3E).

To investigate whether differences in CB2-CysCAR-T cell cytotoxicity could be attributed to variations in surface thiol availability, we quantified exofacial thiol levels across all tested BCL cell lines and compared them to those of healthy PBMCs. All cell lines displayed a significantly higher abundance of exofacial thiols relative to PBMCs (Figure S6). Although slight variations were observed among the cancer cell lines, these did not correlate with the differences in cytotoxicity shown in Figure 3E. These findings suggest that all BCL cell lines expose sufficient surface thiols to trigger CysCAR-T cell activation and that the variability in cytotoxic responses likely reflects differences in the intrinsic susceptibility of the cell lines to CAR-T cell-mediated killing.

In conclusion, CB2-CysCAR-T cells demonstrate potent activity against multiple BCL subtypes, including antigen escape models and BCL cells resistant to conventional CD19-CAR-T cell therapy, highlighting their broad and antigen-independent targeting capability.

### Cysteine-Engineering of CAR-T Cells Overcomes Anti-CD19 CAR-T Therapy Resistance in BCL and Holds Transformative Potential for the Treatment of Solid Tumors

Encouraged by our results, we investigated whether cysteine engineering could enhance the activity of Kymriah. We generated bifunctional CD19-CysCAR-T cells designed to simultaneously target CD19 and cancer-associated extracellular redox states. As CD19-directed CAR-T cells are based on the scFv FMC63 with two domains in contrast to single-domain nanobodies, we tested various cysteine mutations to identify the optimal position within the three-dimensional structure: two serine residues (S190 and S229), a glycine residue (G228), an asparagine residue (N92), or an aspartic acid residue (D156) (Figure 4A,B, hereafter CD19-CysCAR). All generated CD19-CysCAR constructs achieved transduction efficiencies exceeding 50%, except for G228C, which showed a reduced efficiency, presumably due to impaired binding of the detection agent (Figure S7A). Nevertheless, ^G228C^CD19-CysCAR-T cells exhibited detectable binding to recombinant, fluorescently labeled CD19 compared to nontransduced T cells, confirming functional CAR expression (Figure S7B).

**Figure 4:**
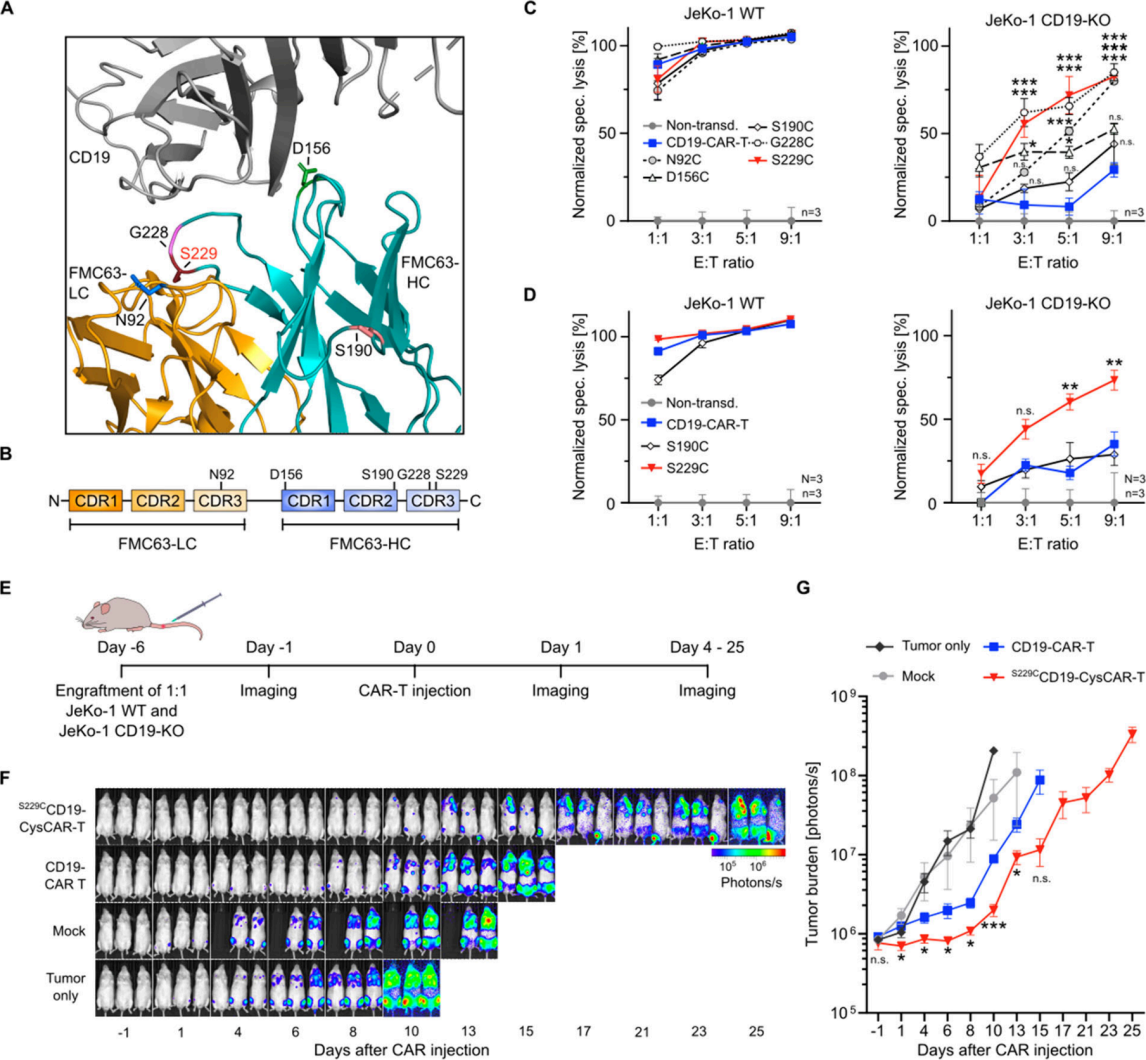
Bifunctional activity of anti-CD19 CysCAR-T cells. (**A**) Crystal structure of FMC63 in complex with CD19 shows the cysteine-mutated residues N92, D156, S190, G228, and S229 (PDB-ID: 7URV). (**B**) Light (LC) and heavy chain (HC) domains of FMC63 and cysteine-mutated residues. (**C**) Specific lysis of BCL cells after co-incubation with CAR-expressing T cells and (**D**) pooled data for three donors (*N* = 3). (**E**) Schedule of CAR-T treatment in mice bearing 1:1 JeKo-1 WT and CD19KO. (**F**) BLI images of the tumor burden in mice (*n* = 3). (**G**) Quantification of tumor burden. Data are shown as mean ± SEM of (**C**) *n* = 3, (**D**) *n* = 3, (**G**) *n* = 3, and *n* = 2 for ‘mock’ group from day 4. Statistical significance was calculated via (**C, D**) two-way ANOVA and Tukey’s *posthoc* and (**G**) multiple unpaired *t* tests: n.s., not significant; *p < 0.05; **p < 0.01, ***p < 0.001.

We compared the cytotoxic activity of conventional and CD19-CysCAR-T cells in our previously established antigen escape model with JeKo-1 CD19KO cells using a luciferase-based cytotoxicity assay. All cysteine-engineered CD19-CysCAR-T cells targeted JeKo-1 WT with efficiencies similar to conventional CAR-T cells, indicating preserved CD19 affinity and proper CAR expression across all cysteine mutants (Figure 4C, left panel). Notably, only two CD19-CysCAR-T cells with structurally adjacent cysteine mutations (G228C and S229C) showed an additional activity against CD19-negative lymphoma (JeKo-1 CD19KO), whereas conventional CD19-CAR-T cells showed no effect (Figure 4C, right panel). Moreover, ^S229C^CD19-CysCAR-T cells derived from three independent healthy blood donors consistently demonstrated cytotoxicity against a therapy-resistant BCL model (*N* = 3, *n* = 3), highlighting their clinical potential (Figure 4D).

To establish a universal and adaptable framework for next-generation CysCAR-T therapies, we aimed to identify the key structural differences correlating with the cytotoxic activity observed among CD19-CysCAR-T cells. For this, we analyzed the FMC63-CD19 crystal structure (PDB: 7URV),^35^ calculating relative solvent-accessible surface area (SASA) and minimal distances to CD19 for each mutated residue. At a 9:1 E:T ratio, mutations with lower solvent accessibility (SASA < 0.5) correlated with higher cytotoxicity, suggesting that less solvent-exposed residues enhance CAR function (Table S1). Interestingly, residues with an increased relative SASA value appeared to be less active against CD19-negative lymphoma, supposedly due to increased spontaneous oxidation.^36^ Namely, residues close to CD19 (4–8 Å for S229C, G228C, D156C, and N92C) yielded greater cytotoxic effects, while the more distant S190C mutation (16.7 Å from CD19) showed no significant activity (Figure 4C, right panel; Table S1). Thus, these structural insights can serve as a basis for cysteine engineering of future CAR-T constructs.

To explore the feasibility and potential of dual-targeting anti-CD19 CysCAR-T immunotherapy in overcoming antigen escape, we established a murine CD19-antigen escape model. Mice co-engrafted with equal numbers of JeKo-1 WT and CD19-KO cells were treated with mock T cells, conventional CD19-CAR-T cells, or ^S229C^CD19-CysCAR-T cells (*n* = 3 per group). ^S229C^CD19-CysCAR-T cells delayed tumor growth beyond 25 days and extended survival compared to conventional CD19-CAR-T cells (Figure 4E–G). No significant changes in body weight (Figure S9) or visible signs of systemic toxicity were observed in any treatment group. However, tumor growth persisted, albeit delayed, indicating the need for further optimization to improve the *in vivo* efficacy.

Taken together, these results demonstrate that thiol-mediated anti-CD19 CysCAR-T immunotherapy outperforms conventional CD19-CAR-T therapy by targeting both CD19 and the abnormal surface redox states in the BCL. This approach was safe and effective in a murine model, supporting its potential for overcoming therapy resistance. However, given the small sample size in our murine study (*n* = 3 per group), further investigation with larger cohorts is required. *In vivo* efficacy studies using a lower proportion of knockout cells or patient-derived xenograft models will more accurately emulate clinical conditions where antigen escape arises under treatment pressure, which may give further insight into the therapeutic durability of CysCAR-T cells and the prevention of resistance formation.

As CB2 binds specifically to breast cancer but not to nontumorigenic breast cells,^23^ we wished to demonstrate the potential applications of CysCAR-T cells to solid tumors. Therefore, we performed preliminary coculture experiments using CB2- and CD19-CAR-T cells against the human triple-negative breast cancer cell line MDA-MB-231 and the healthy human breast epithelial cell line MCF 10A. As expected, the cysteine-engineered CB2-CysCAR and ^S229C^CD19-CysCAR-T cells exhibited significant, concentration-dependent cytotoxicity toward MDA-MB-231 cells while sparing MCF 10A (Figure S10). These findings suggest that redox-reactive CAR-T cells may also be effective against breast cancer, supporting the further exploration of cysteine engineering for breast-cancer-specific CAR-T cell therapies. Moreover, these results underscore the redox specificity of the approach. Even though MDA-MB-231 cells do not express CD19, they were still targeted by S229CCD19-CysCAR-T cells, emphasizing the key role of the single cysteine modification. The lack of cytotoxicity against healthy epithelial cells further highlights the cancer-specific activity of CysCAR-T cells.

## Conclusions

Despite the transformative impact of CAR-T cell therapy in treating CD19-positive hematological cancers,^1^ significant challenges remain, particularly due to the ability of cancers to adapt to therapy by antigen escape.^4,37^ Here, we present bifunctional CysCAR-T cells, which exhibit enhanced anticancer activity compared to conventional anti-CD19 CAR-T cells. These cells are designed to directly interact with the abnormal extracellular redox environment of BCL, thereby providing an additional layer of targeting specificity. We show that the cytotoxic activity of bifunctional CysCAR-T cells against BCL cancer cells is comparable to that of conventional anti-CD19 CAR-T cells. However, a single cysteine residue introduced in the CAR construct is sufficient to allow CysCAR-T cells to overcome antigen escape *in vitro*, and in the pilot CD19KO murine study by targeting the altered redox state rather than relying solely on a single surface antigen. Thus, thiol-mediated CysCAR-T therapy might become a promising strategy for patients with relapsed or refractory BCL who do not respond to conventional CAR-T therapy.

In future experiments, we will investigate the details of the molecular mechanisms underlying the activation of CysCAR-T cells. For example, cell–cell interactions may involve a dynamic covalent exchange reaction between reactive cysteine thiols on the CARs and thiols or disulfides on the surface of cancer cells.^32,38^ Moreover, *cis*-interactions between adjacent reactive free thiols may form disulfides on the CAR, which might then interact with reactive thiols on the BCL cell surface. Understanding these mechanisms is key to assessing whether employing reducing agents to unpair newly formed disulfides before *in vivo* application could help to enhance the cytotoxicity of CysCAR-T cells. Notably, it is also plausible that mice are not an optimal model for our *in vivo* investigation. In contrast to subcutaneous or orthotopic injection of cancer cells in mouse xenografts of solid tumors, *in vivo* blood cancer studies require BCL cells to be introduced through intravenous injection to mimic systemic spread. Thus, further investigation will be necessary to rule out possible neutralization of the CysCAR-T cells or their targets on the cell surface of injected cancer cells by blood-borne enzymatic and nonenzymatic antioxidants that protect cells from oxidative stress.

Off-tumor effects on healthy tissues by exposing free exofacial thiols could present a potential limitation of the redox-specific mechanism employed by CysCAR-T cells. Under physiological conditions, the cell surface typically exhibits a more oxidized redox state compared to the intracellular environment.^16^ However, certain conditions, such as T cell activation or viral infections, can shift the extracellular milieu toward a more reduced state.^39–42^ Despite this, our *in vitro* experiments with healthy cells and *in vivo* data suggest that, under nonpathological conditions, surface thiol levels on healthy cells remain below the activation threshold required for CysCAR-T cell engagement. Consistent with this, several studies have demonstrated the successful (non-CAR-T) targeting of altered cancer redox states *in vivo* without significant systemic effects, highlighting the therapeutic potential of this approach.^43–46^

We envision the broader applicability of the CysCAR-T approach, extending beyond hematological malignancies to other challenging cancers with complex microenvironments and mechanisms of immune evasion.^47^ In breast cancer, malignant cells upregulate the expression of protein-disulfide isomerases (PDIs) and display higher levels of cell surface thiols.^25,48^ This phenotype was also previously verified by CB2 binding to breast cancer cell lines.^23^ Our preliminary cytotoxicity assays on breast cancer cells demonstrate the potential of CysCAR-T cells for breast cancer treatment and underscore the redox-specific nature of this approach, which relies solely on a single cysteine modification. Thus, a redox-guided CysCAR-T cell approach not only enables precise tumor targeting but also offers a new solution to overcome BCL antigen escape, one of the most limiting factors of conventional CAR-T cell therapy. These findings open the door to a new class of bifunctional CAR-T therapies that sense both specific antigens and altered redox states of cancer cells with the potential to reshape treatment strategies across diverse malignancies.

## Supplementary Material



## Notes

Detailed materials and methods; supplementary Figures S1–S10 showing additional data on the CAR constructs used in this study (Figure S1), the sorting efficiency of CB2-CD28-CAR_GFP and GFP control T cells (Figure S2), the gating strategy of flow cytometry-based apoptosis assays (Figure S3), the purification of recombinant nanobodies LaG-16 and ^S106C^LaG-16 (Figure S4), the knockout efficiencies of JeKo-1 cell lines (Figure S5), the quantification of surface thiols on BCL cell lines and healthy human PBMCs (Figure S6), the transduction efficiencies of WT and mutated CD19-CAR-T cells (Figure S7), the validation of firefly luciferase-expressing SC-1 cells (Figure S8), mouse body weight data (Figure S9), and CysCAR-T cell targeting of breast cancer cells (Figure S10); and supplementary Table S1 showing structural parameters of CD19-CAR mutants and corresponding cytotoxic activity (PDF)

## #

Copenhagen Center for Glycomics, University of Copenhagen, 1172 Copenhagen, Denmark
