# Supplementary material for: Bifunctional Cysteine-Engineered CAR‑T Cells Enable Thiol-Mediated Targeting to Overcome Antigen Escape in B Cell Lymphoma
Source: ACS Cent Sci. 2025 Aug 7;11(10):1852–61. doi: 10.1021/acscentsci.5c00816 (PMC12550615; doi:10.1021/acscentsci.5c00816)
Supplement: Supplementary file 1 [file oc5c00816_si_001.pdf]

Supporting Information

Bifunctional Cysteine-Engineered CAR-T cells Enable Thiol-Mediated Targeting to Overcome Antigen Escape in B Cell Lymphoma

Jost Lühle[1,2], Simon Krost[3,4,5], Felix Goerdeler[1,2†], Aina Valentí[1,2], Elena Shanin[1], Christian Seitz[3,4,5], Peter H. Seeberger,[1,2], Oren Moscovitz[1*]

[1]Department of Biomolecular Systems, Max Planck Institute of Colloids and Interfaces; Potsdam, Germany.
[2]Institute of Chemistry and Biochemistry, Freie Universität Berlin; Berlin, Germany.
[3]Department of Pediatric Hematology and Oncology, University of Tübingen; Tübingen, Germany.
[4]Hopp-Children's Cancer Center Heidelberg (KiTZ); Heidelberg, Germany.
[5]Department of Pediatric Oncology, Hematology and Immunology, Heidelberg University Hospital; Heidelberg, Germany.
[†]Present address: Copenhagen Center for Glycomics, University of Copenhagen; Copenhagen, Denmark.
[*]Correspondence to: oren.moscovitz@mpikg.mpg.de (O.M.)

Contents

SUPPLEMENTARY MATERIALS AND METHODS **2**
- **Protein Expression and Structural Analysis** **2**
- **Cell Lines** **2**
- **CAR Construct Design and Cloning** **2**
- **Lentiviral Packaging and CAR-T Cell Generation** **2**
- **Flow Cytometry** **3**
- **Determination of surface thiol levels** **3**
- **Apoptosis Assay** **3**
- **Cytotoxicity Assay** **4**
- **ELISA** **4**
- **Animal Models** **4**
- **Ethics Statement** **4**

SUPPLEMENTARY FIGURES **5**
SUPPLEMENTARY TABLES **10**
SUPPLEMENTARY REFERENCES **10**

SUPPLEMENTARY MATERIALS AND METHODS

Protein Expression and Structural Analysis

CB2 and ^{C105S}CB2 were expressed and purified as described elsewhere.[1] Nanobodies (LaG-16, ^{S106C}LaG-16) were transformed in *E.coli* ArcticExpress (DE3) cells (Agilent Technologies, Santa Clara, CA) using plasmid pET21-pelB-LaG-16 (plasmid was a gift from Michael Rout, Addgene plasmid #172746; http://n2t.net/addgene:172746; RRID:Addgene_172746)[2] and expressed in LB medium with 1 mM isopropyl-β-D-1-thiogalactopyranoside at 12°C overnight. Cells were lysed with 5 freeze-thaw cycles, alternating between 37°C water bath and dry ice. Nanobodies were purified by Nickel-NTA (ThermoFisher Scientific, Waltham, MA) and size exclusion chromatography using ÄKTApurifier S200 column (Cytiva, Marlborough, MA), and validated by SDS-PAGE (Fig. S4).

FMC63-CD19 structure (PDB ID: 7URV)[3] was analyzed in PyMOL for solvent-accessible surface area (SASA)[4] and antigen distance. SASA values were computed via the command ‘PyMOL>get_sasa_relative polymer’. The minimal antigen distance was assessed using wizard tool in PyMOL between the amino acid side chains and the closest atom of CD19.

Cell Lines

SC-1 and JeKo-1 cells were cultured in RPMI 1640 with 10% FCS, 2 mM L-glutamine, 1 mM pyruvate and 100 U/mL Penicillin-Streptomycin (PAN-Biotech, Aidenbach, Germany). Luciferase/GFP-expressing cells were generated by lentiviral transduction and sorted using FACSMelody™ Cell Sorter (BD, Franklin Lakes, NJ). MDA-MB-231 cells were cultured in DMEM + 10 % FCS + 2 mM glutamine + 1x penicillin/streptomycin. MCF 10A cells were cultured in DMEM/F12 (1:1) + 5 % horse serum + 20 ng/mL EGF + 0.5 mg/mL hydrocortisone + 100ng/mL cholera toxin + 10 μg/mL insulin + 2 mM glutamine + 1x penicillin/streptomycin. All cell lines were tested negative for mycoplasma contamination monthly.

CAR Construct Design and Cloning

Second-generation CAR constructs (CB2-CD28-CAR, C105SCB2-CD28-CAR, LaG-16-CD28-CAR, CB2-41BB-CAR, FMC63 mutants N92C, D156C, S190C, G228C, S229C) were designed with domains including human EF-1α promoter, signal peptides (SP), antibody fragments (CB2, LaG-16, FMC63), hinges (IgG1-Fc or CD8), transmembrane domains (CD28 or CD8), costimulatory domains (CD28 or 4-1BB), CD3ζ signaling domain (SD), and Woodchuck Hepatitis Virus Posttranscriptional Regulatory Element (WPRE) or for CB2-CD28-CAR_GFP construct additionally with human cytomegalovirus enhancer and promotor (CMV) and green fluorescent protein (GFP). Constructs were synthesized by VectorBuilder Inc. (Chicago, IL), IDT (Coralville, IA), cloned using restriction digestion (e.g. XbaI, KpnI, EcoRV, NdeI) and ligation or NEBuilder® HiFi DNA Assembly (NEB, Ipswich, MA). GFP was removed from CB2-CD28-CAR_GFP using PCR and restriction cloning; C105SCB2 mutant was generated by overlap extension PCR as described by Heckman et al.[5]

Lentiviral Packaging and CAR-T Cell Generation

HEK293T cells were cultured in DMEM with 10% FCS, 2 mM L-glutamine and 100 U/mL Penicillin-Streptomycin (PAN-Biotech, Aidenbach, Germany). HEK293T cells were transfected with pLV-CAR, psPAX2 (plasmid was a gift from Didier Trono, Addgene plasmid # 12260; http://n2t.net/addgene:12260; RRID:Addgene_12260) or pMD2.G (plasmid was a gift from Didier Trono, Addgene plasmid # 12259; http://n2t.net/addgene:12259; RRID:Addgene_12259) using TransIT 293T (Mirus Bio, Madison, WI). As described by Jiang et al.,[6] lentiviral particles were harvested at 1,500 g for 10 min after 48 h and concentrated by sucrose cushion (50 mM Tris-HCl pH 7.4, 100 mM NaCl, 0.5 mM EDTA, 10% w/v sucrose)

centrifugation at 4:1 v/v ratio at 10,000 g, 4°C for 4 h, resuspended in PBS and stored in 500 µL aliquots at -80°C.
Human PBMCs (German Red Cross, Berlin, Germany) were isolated using SepMate™-50 tubes (Stemcell Technologies, Vancouver, Canada) and T cells were enriched with Pan T Cell Isolation Kit (Miltenyi Biotec, Bergisch Gladbach, Germany). $1x10^6$ T cells in RPMI 1640, 10% FCS, 2 mM L-glutamine, 1 mM pyruvate, 100 U/mL penicillin-streptomycin and 30 U/mL human IL-2 (Peprotech Inc., Cranbury, NJ) were activated with 25 µL Dynabeads™ CD3/CD28 (ThermoFisher Scientific, Waltham, MA) and transduced with lentivirus (1:15, 5 µg/mL polybrene) and sorted for GFP expression using FACSMelody™ Cell Sorter (BD, Franklin Lakes, NJ).
Luciferase- and GFP-expressing SC-1 cells were generated using plasmid SIN40C.SFFV.Luciferase.IRES.GFP as described above (plasmid was a gift from Jan-Henning Klusmann, Addgene plasmid # 169308; http://n2t.net/addgene:169308; RRID:Addgene_169308).[7] Briefly, $1x10^6$ SC-1 cells were transduced with 120 µL concentrated lentivirus and 8 µg/mL polybrene and sorted for GFP^+ on day 8. Clones were screened on day 23 for uniform GFP and CD19 expression by flow cytometry with anti-human CD19-PE (1:100, Fig. S8). JeKo-1 cell expressing luciferase were generated as described previously.[8]

Flow Cytometry

Flow cytometry was conducted using FACSCanto™ II Flow Cytometry System (BD, Franklin Lakes, NJ) to assess nanobody binding to SC-1 cells and CAR-T cell activation upon co-culture with SC-1 cells. For nanobody binding, SC-1 cells ($1x10^6$ cells/sample) were incubated with 24 µM nanobody or PBS (1 hour, RT), stained with MonoRab™ iFluor 647 (1 hour, RT), and analyzed.
For T cell activation, non-transduced, GFP+ and CB2-CAR-T cells were co-cultured with SC-1 cells (E:T ratio 9:1, n=3, 72 h), stained with PE/Cy7 anti-human CD25, CD107a (LAMP-1) or CD69 and APC-CD3 (BioLegend, San Diego, CA), and quantified against controls (T cell only) using one-way ANOVA with Tukey's post-hoc test.
For transduction efficiencies, nanobody-based and anti-CD19 CAR-T cells were stained with MonoRab™ iFluor 647 (1:500, 30 min, 4°C; GenScript, Piscataway, NJ) and CD19-Fc chimera Alexa Fluor® 647 (1:500, 30 min, RT; Bio-Techne, Minneapolis, MN), respectively. FMC63 mutants CAR-T cells were stained with anti-idiotypic FITC-FMC63 scFv (1:50; ACROBiosystems, Newark, DE). Transduction efficiencies were assessed using FACSCanto™ II (BD, Franklin Lakes, NJ).
For knockout efficiencies, WT, CD19 and CD20 JeKo-1 cells were stained with anti-human CD19-PE and CD20-APC (both 1:100; BioLegend, San Diego, CA) for 1 hour at RT (Figure S5).

Determination of surface thiol levels

One million PBMCs or BCL cells were pelleted and incubated with Alexa Fluor™ 647-labeled maleimide (1:1,000, Jena Bioscience) or PBS for 15 min on ice in the dark. Cells were washed thrice with PBS and analyzed by flow cytometry. The median fluorescence intensity (MFI) from three independent experiments was quantified. Differences between cell lines were tested for significance via one-way ANOVA and Tukey's post-hoc tests.

Apoptosis Assay

CAR-T cells co-cultured with $5x10^3$ SC-1 or PBMCs (E:T 1:1 to 9:1, n=3, 72 h) were stained with anti-human CD3-APC (1:200; clone OKT3; Biolegend, San Diego, CA), Annexin V-PE (1:100; BioLegend, San Diego, CA) and 7-AAD (1:100; ThermoFisher Scientific, Waltham, MA) in Annexin V Binding Buffer (BioLegend, San Diego, CA) at RT, 15 min and analyzed by flow cytometry. Target cells were gated on as $CD3^-/GFP^-$. PBMCs were stained with

CellTrace™ Far Red Cell Proliferation Kit (ThermoFisher Scientific, Waltham, MA) and gated on as $CellTrace™^{+}/GFP^{-}$. Statistical significance was calculated via two-way ANOVA and Tukey's post-hoc tests.

Cytotoxicity Assay

Non-transduced and CAR-expressing T cells were co-cultured with $5x10^3$ luciferase-expressing SC-1 or JeKo-1 cells at E:T ratios 1:1, 3:1, 5:1, and 9:1 (n=3) at 37°C for 24 h (SC-1) or 48 h (JeKo-1). As control for cell lysis, BCL cells were cultured alone. T cell numbers were adjusted to normalize CAR-expressing T cells across constructs. For assays with breast cancer cells, T cells were incubated with $3x10^4$ luciferase-expressing MDA-MB-231 or non-tumorigenic MCF 10A cells at E:T ratios 0.3:1, 0.8:1, 2.5:1, and 7.5:1 (n=2) at 37°C for 24 h.

After coculture, 100 µg/mL D-luciferin potassium salt (Abcam, Cambridge, UK) was added to cells with 1% NP-40 included in lysis controls and incubated at 37°C for 1 hour. Relative light units (RLU) were measured in a CLARIOstar Plus plate reader (BMG LABTECH, Ortenberg, Germany) with a 10-second exposure per well. Normalized specific lysis was calculated as:

$$\text{Normalized specific lysis [\%]} = 100 \times \frac{\text{NT RLU} - \text{test RLU}}{\text{NT RLU} - \text{lysis control RLU}}$$

NT is RLU from non-transduced T cell cocultures. Data from independent experiments was pooled by calculating the grand mean with pooled standard error and statistical significance was calculated via two-way ANOVA and Tukey's post-hoc tests.

ELISA

IFN-γ and TNF-α were determined for CAR-T cell co-cultures with SC-1 cells, PBMCs or RBCs (9:1 ratio, n=3) using human IFN-γ and TNF-α mini TMB ELISA Kits (Peprotech Inc., Cranbury, NJ) or human IFN-γ ELISA Kit (ThermoFisher Scientific, Waltham, MA). Statistical significance was calculated via two-way ANOVA and Tukey's post-hoc tests.

Animal Models

In vivo experiments were conducted as described elsewhere.[9] Briefly, 2- to 12-month-old male NSG™ mice (Charles River Laboratories) were engrafted with a 1:1 mixture of JeKo-1 WT Luc/GFP and JeKo-1 CD19-KO Luc/GFP cells (0.5×10^6) on day −6, followed by 3×10^6 CAR-T cells on day 0. Tumor burden was monitored using bioluminescence IVIS imaging spectrum (Perkin Elmer, Waltham, MA) with 1 s, 10 s, 20 s exposure times, analyzed using Living Image Software 4 (Perkin Elmer, Waltham, MA). Statistical significance was calculated via multiple unpaired t tests. Additionally, mice body weight was measured at each time point of bioluminescence imaging.

Ethics Statement

Human blood sampling was approved by Max Planck Society (2021_08). Animal studies adhered to FELASA guidelines at University Clinic Tübingen.

SUPPLEMENTARY FIGURES

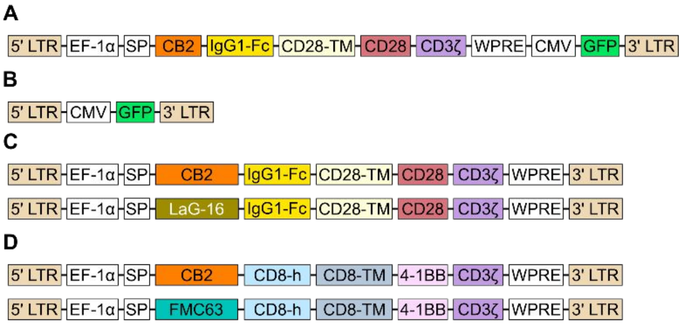

Fig. S1: Overview of CAR constructs used in this study. A, CB2-CD28-CAR_GFP construct. **B**, GFP control construct. **C**, CD28-CAR constructs. **D**, 41BB CAR constructs. LTR – long terminal repeat, EF-1α – human elongation factor-1 alpha promotor, SP – signal peptide, IgG1-Fc – human IgG1-Fc hinge, CD28-TM – CD28 transmembrane domain, CD28 – CD28 costimulatory domain, CD3ζ – CD3ζ signaling domain, WPRE – Woodchuck Hepatitis Virus Posttranscriptional Regulatory Element, CMV – human cytomegalovirus enhancer and promotor, GFP – green fluorescent protein, CD8-h – CD8 hinge, CD8-TM – CD8 transmembrane domain, 4 1BB – 4 1BB costimulatory domain.

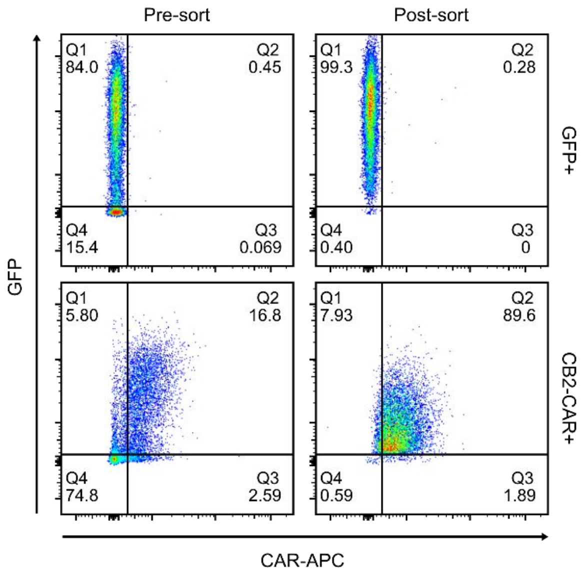

Fig. S2: Sorting efficiency of CB2-CD28-CAR_GFP and GFP control T cells. Pre-sorting (left panels) and post-sorting (right panels) efficiencies were measured via flow cytometry. CAR-APC and GFP signals were assessed.

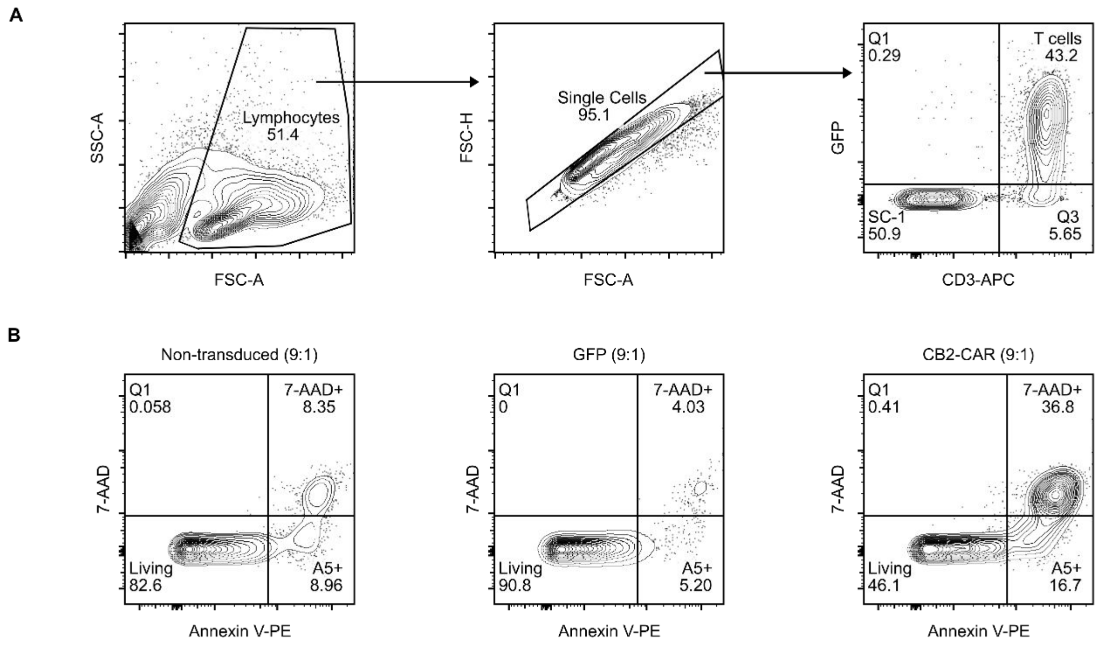

Fig. S3: Gating strategy for flow cytometry-based apoptosis assays. A, Gating of single target cells (SC-1). Cells were gated for lymphocytes (left panel), single cells (middle panel), and SC-1 cells (GFP-/CD3+; right panel). **B**, Gating of apoptotic target cells. Single SC-1 cells (gated in A) were further gated for Annexin V-PE+/7-AAD- cells (early apoptotic cells) and Annexin V-PE+/7-AAD+ cells (late apoptotic cells). Example for effector to target ratio of 9:1 is shown. FSC – forward scatter, SSC – side scatter.

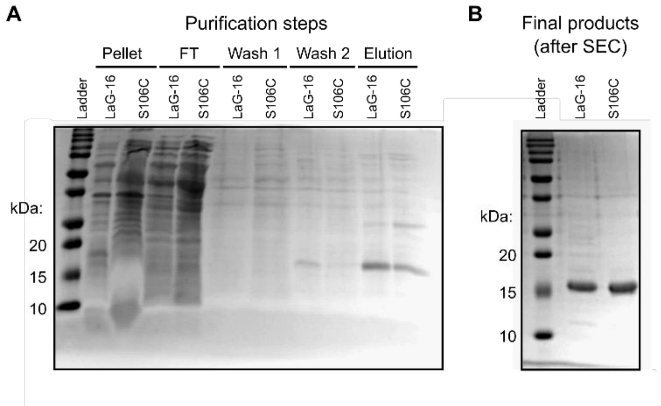

Fig. S4: Purification of recombinant nanobodies LaG-16 and ^{S106C}LaG-16 from Escherichia coli. A, Sodium dodecyl sulfate poly-acrylamide gel electrophoresis (SDS-PAGE) of purification steps. Samples from the different steps of Nickel-NTA affinity chromatography are shown. Nanobodies are visible in the gel at a size of approximately 15 kilodalton. **B**, Purity of final products. FT – flow through, kDa – kilodalton, SEC – size exclusion chromatography.

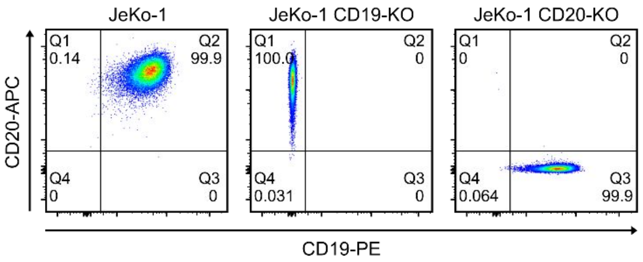

Fig. S5: Knockout efficiencies of JeKo-1 cell lines. Efficiencies were determined via flow cytometry. Gates were adjusted according to non-stained control (not shown). Left panel: JeKo-1, middle panel: JeKo-1 CD19-knockout, right panel: JeKo-1 CD20-knockout.

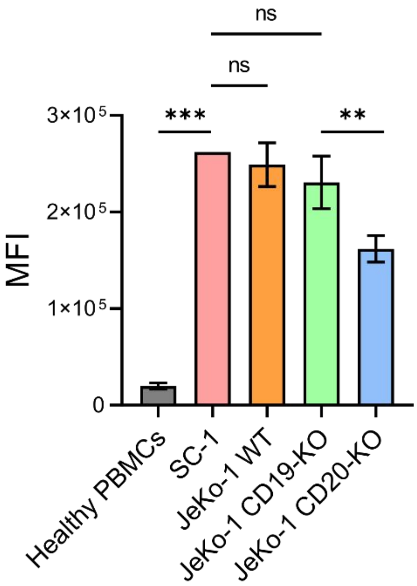

Fig. S6: Quantification of surface thiols on BCL cell lines and healthy human PBMCs. The relative amount of free surface thiols was measured via flow cytometry after incubating the cells with Alexa Fluor™ 647-labeled maleimide. Median fluorescence intensities (MFI) from three independent experiments are shown as mean ± SEM. Statistical significance was calculated via one-way ANOVA and Tukey's post-hoc tests: n.s. – not significant; * – $p<0.05$; ** – $p<0.01$; *** – $p<0.001$.

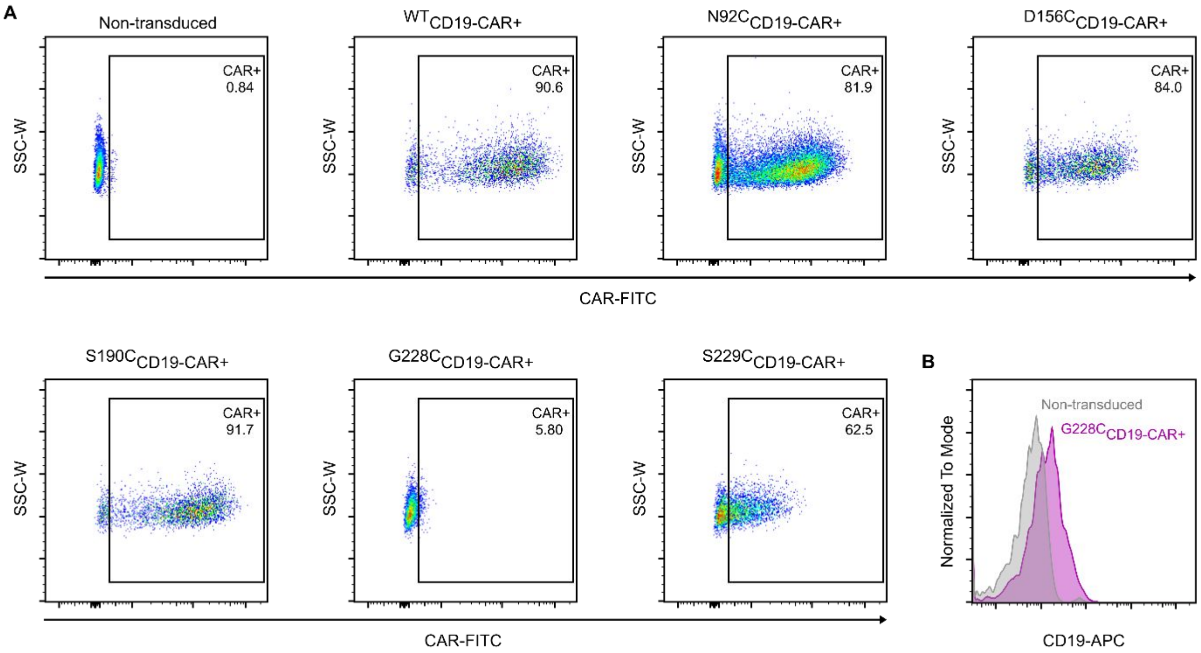

Fig. S7: Transduction efficiencies of WT and mutated CD19-CAR-T cells. A, Determination of transduction efficiencies of CD19-CAR variants on day 6 after transduction via flow cytometry. Representative plots are shown. Percentages of CAR+ T cells are indicated in the graphs. **B**, Verification of CAR expression on G228CCD19-CAR-T cells via staining with fluorescent CD19 (flow cytometry).

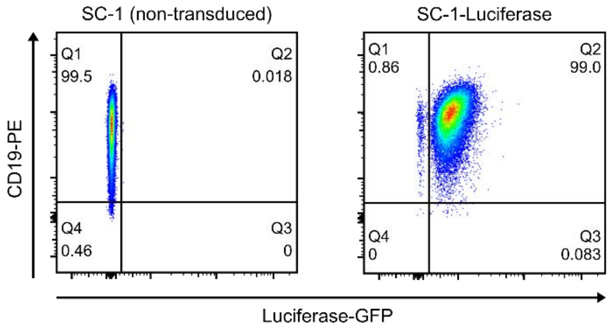

Fig. S8: Validation of firefly luciferase-expressing SC-1 cells. SC-1 cell line was transduced with lentivirus carrying firefly luciferase and GFP. CD19 and Luciferase-GFP expression was assessed via flow cytometry to confirm successful generation of luciferase-expressing SC-1 cells.

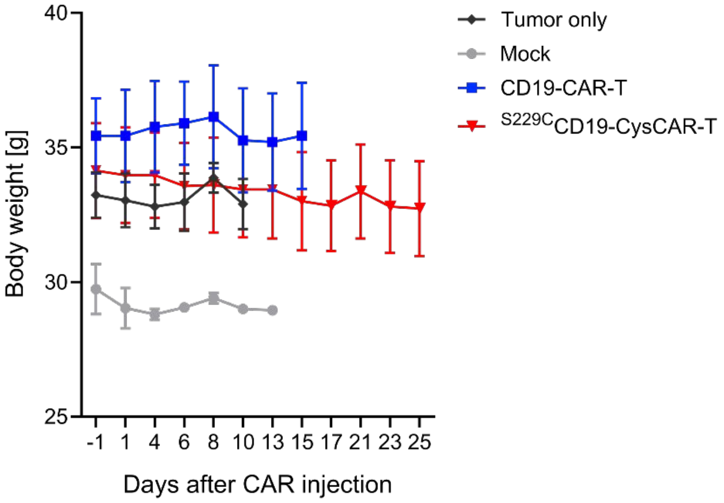

Fig. S9: Mice body weight data from *in vivo* experiment. The body weight of the mice was measured at each time point of the experiment, shown in Figure 4E–G. Data are shown as mean ± SEM.

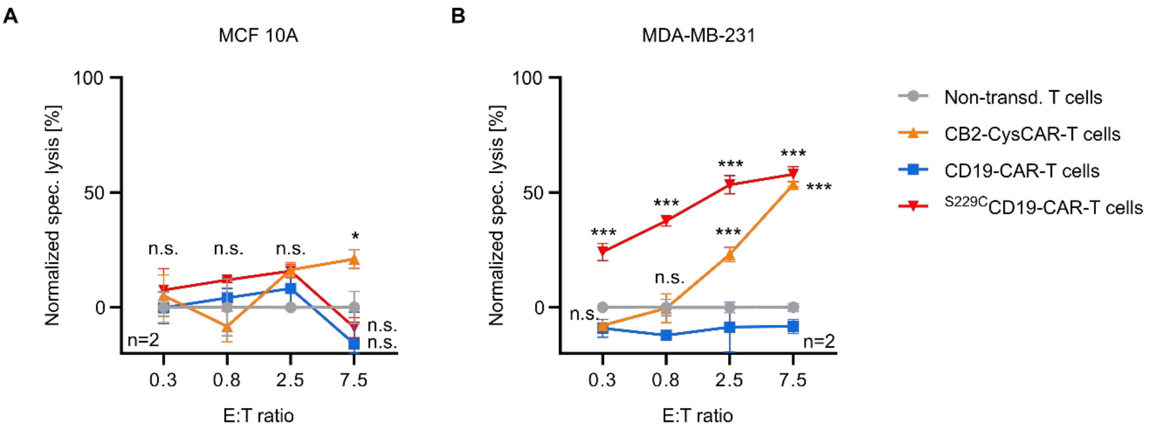

Fig. S10: CysCAR-T cells efficiently target breast cancer cells but not healthy human breast epithelial cells. Specific lysis of healthy human breast epithelial cell line MCF 10A (**A**) and human breast cancer cell line MDA-MB-231 (**B**) after coincubation with different CAR-T cells. Data are shown as mean ± SEM (n=2). Statistical significance was calculated via two-way ANOVA and Tukey's post-hoc tests: n.s. – not significant; * – $p<0.05$; ** – $p<0.01$; *** – $p<0.001$.

SUPPLEMENTARY TABLES

Table S1: Structural parameters of CD19-CAR mutants and corresponding cytotoxic activity. Specific lysis values refer to co-cultures against JeKo-1 CD19-KO at the effector to target (E:T) ratio of 9:1 (shown in Fig. 4C).

CD19-CAR mutant	Specific lysis at 9:1 E:T ratio [%]	Relative solvent-accessible surface area	Minimal distance to antigen [Å]
G228C	85	0.30	4.2
S229C	83	0.03	7.9
N92C	80	0.34	5.0
D156C	53	0.82	5.7
S190C	44	0.93	16.7

SUPPLEMENTARY REFERENCES

(1) Goerdeler, F.; Reuber, E. E.; Lühle, J.; Leichnitz, S.; Freitag, A.; Nedielkov, R.; Groza, R.; Ewers, H.; Möller, H. M.; Seeberger, P. H.; Moscovitz, O. Thiol-Mediated Uptake of a Cysteine-Containing Nanobody for Anticancer Drug Delivery. *ACS Cent. Sci.* **2023**, *9* (6), 1111–1118.

(2) Fridy, P. C.; Li, Y.; Keegan, S.; Thompson, M. K.; Nudelman, I.; Scheid, J. F.; Oeffinger, M.; Nussenzweig, M. C.; Fenyö, D.; Chait, B. T.; Rout, M. P. A Robust Pipeline for Rapid Production of Versatile Nanobody Repertoires. *Nat. Methods* **2014**, *11* (12), 1253–1260.

(3) He, C.; Mansilla-Soto, J.; Khanra, N.; Hamieh, M.; Bustos, V.; Paquette, A. J.; Angus, A. G.; Shore, D. M.; Rice, W. J.; Khelashvili, G.; Sadelain, M.; Meyerson, J. R. CD19 CAR Antigen Engagement Mechanisms and Affinity Tuning. *Sci. Immunol.* **2023**, *8* (81), 1–13.

(4) Ali, S.; Hassan, M.; Islam, A.; Ahmad, F. A Review of Methods Available to Estimate Solvent-Accessible Surface Areas of Soluble Proteins in the Folded and Unfolded States. *Curr. Protein Pept. Sci.* **2014**, *15* (5), 456–476.

(5) Heckman, K. L.; Pease, L. R. Gene Splicing and Mutagenesis by PCR-Driven Overlap Extension. *Nat. Protoc.* **2007**, *2* (4), 924–932.

(6) Jiang, W.; Hua, R.; Wei, M.; Li, C.; Qiu, Z.; Yang, X.; Zhang, C. An Optimized Method for High-Titer Lentivirus Preparations without Ultracentrifugation. *Sci. Rep.* **2015**, *5*, 13875.

(7) Alejo-Valle, O.; Weigert, K.; Bhayadia, R.; Ng, M.; Issa, H.; Beyer, C.; Emmrich, S.; Schuschel, K.; Ihling, C.; Sinz, A.; Zimmermann, M.; Wickenhauser, C.; Flasinski, M.; Regenyi, E.; Labuhn, M.; Reinhardt, D.; Yaspo, M.-L.; Heckl, D.; Klusmann, J.-H.; Hematology, P. The Megakaryocytic Transcription Factor ARID3A Suppresses Leukemia Pathogenesis. *Blood* **2022**, *139* (5), 651–665.

(8) Seitz, C. M.; Mittelstaet, J.; Atar, D.; Hau, J.; Reiter, S.; Illi, C.; Kieble, V.; Engert, F.; Drees, B.; Bender, G.; Krahl, A. C.; Knopf, P.; Schroeder, S.; Paulsen, N.;

Rokhvarguer, A.; Scheuermann, S.; Rapp, E.; Mast, A. S.; Rabsteyn, A.; Schleicher, S.; Grote, S.; Schilbach, K.; Kneilling, M.; Pichler, B.; Lock, D.; Kotter, B.; Dapa, S.; Miltenyi, S.; Kaiser, A.; Lang, P.; Handgretinger, R.; Schlegel, P. Novel Adapter CAR-T Cell Technology for Precisely Controllable Multiplex Cancer Targeting. *Oncoimmunology* **2021**, *10* (1), e2003532-2–16.

(9) Atar, D.; Ruoff, L.; Mast, A. S.; Krost, S.; Moustafa-Oglou, M.; Scheuermann, S.; Kristmann, B.; Feige, M.; Canak, A.; Wolsing, K.; Schlager, L.; Schilbach, K.; Zekri, L.; Ebinger, M.; Nixdorf, D.; Subklewe, M.; Schulte, J.; Lengerke, C.; Jeremias, I.; Werchau, N.; Mittelstaet, J.; Lang, P.; Handgretinger, R.; Schlegel, P.; Seitz, C. M. Rational Combinatorial Targeting by Adapter CAR-T-Cells (AdCAR-T) Prevents Antigen Escape in Acute Myeloid Leukemia. *Leukemia* **2024**, *38*, 2183–2195.
